# Yield prediction by machine learning from UAS-based mulit-sensor data fusion in soybean

**DOI:** 10.1186/s13007-020-00620-6

**Published:** 2020-06-01

**Authors:** Monica Herrero-Huerta, Pablo Rodriguez-Gonzalvez, Katy M. Rainey

**Affiliations:** 1 Department of Agronomy, Purdue University, West Lafayette, IN 47906 USA; 2 Department of Mining Technology, Topography and Structures, Universidad de Leon, Ponferrada, Spain

**Keywords:** Unmanned aircraft system (UAS), High throughput phenotyping, Soybean, Structure from Motion (SfM), Machine learning (ML), Yield, Point clouds

## Abstract

**Background:**

Nowadays, automated phenotyping of plants is essential for precise and cost-effective improvement in the efficiency of crop genetics. In recent years, machine learning (ML) techniques have shown great success in the classification and modelling of crop parameters. In this research, we consider the capability of ML to perform grain yield prediction in soybeans by combining data from different optical sensors via RF (Random Forest) and XGBoost (eXtreme Gradient Boosting). During the 2018 growing season, a panel of 382 soybean recombinant inbred lines were evaluated in a yield trial at the Agronomy Center for Research and Education (ACRE) in West Lafayette (Indiana, USA). Images were acquired by the Parrot Sequoia Multispectral Sensor and the S.O.D.A. compact digital camera on board a senseFly eBee UAS (Unnamed Aircraft System) solution at R4 and early R5 growth stages. Next, a standard photogrammetric pipeline was carried out by SfM (Structure from Motion). Multispectral imagery serves to analyse the spectral response of the soybean end-member in 2D. In addition, RGB images were used to reconstruct the study area in 3D, evaluating the physiological growth dynamics per plot via height variations and crop volume estimations. As ground truth, destructive grain yield measurements were taken at the end of the growing season.

**Results:**

Algorithms and feature extraction techniques were combined to develop a regression model to predict final yield from imagery, achieving an accuracy of over 90.72% by RF and 91.36% by XGBoost.

**Conclusions:**

Results provide practical information for the selection of phenotypes for breeding coming from UAS data as a decision support tool, affording constant operational improvement and proactive management for high spatial precision.

## Background

Estimating morphological plant variables and the non-destructive characterization of traits with high accuracy and cost-effectiveness is imperative for high-throughput phenotyping in precision agriculture [1]. Recent advances in sensor technology provide great opportunities for the use of UAS (Unnamed Aircraft Systems) as a low-cost platform to derive high throughput and precise quantitative phenotyping datasets [2]. This technology offers images at high spatial, temporal and spectral resolution containing precise information about interactions from canopy and solar radiation [3]. Due to the increasing use of UAS, the development of software tools and methodologies to automatically phenotype crops is urgently required. Photogrammetric sensors on board the UAS allow for the application of digital image analysis of cover plant height estimation [4], yield estimation [5], early emergence, senescence rate [6], disease detection [7], quality evaluation [8] and canopy architecture [9]. RGB images have been used to accurately estimate vegetation index by deep neural network [10], while thermal sensors ability to capture canopy temperature has been used to detect water stress [11].

Plant height is a crucial variable connected to stability, yield potential and lodging resistance. This variable has been assessed by UAS as a Structure from Motion (SfM), obtaining high correlations with ground reference measurements for barley [12], wheat [13], poppy [14] and sorghum [15]. In addition, Light Detection and Ranging (LiDAR) is capable of providing 3D data including height and vegetation density areas on canopy structure [16]. It has been used to derive canopy height, fractional cover and above ground biomass [17].

Lately, machine learning (ML) models have been used to model plant traits based on image data. These methods employ sophisticated statistical techniques, being able to approximate complex non-linear functions between image features and biophysical parameters. Concretely, deep learning has been used for temporal phenotype/genotype classification [18]. Moreover, [19] use *k*-NN as a classification method to analyse images of diverse germination phenotypes as well as to detect single seed germination. In addition, geometric parameters such as leaf counting have been addressed through plant models by [20]. The best characteristic of ML is the limited prior information necessary for it to be applied. This is due to these model’s ability to capture assumptions and essential distributions directly from the training dataset [21]. Thus, the effect of the unknown variability is significantly reduced. As a disadvantage, the over-fitting of the models is a continuing problem that is difficult to mitigate [22]. Another weak point common in ML is the necessity for a similar distribution between training and testing datasets so that the model has the ability to properly predict variables; even for extensive training data. When distribution differences between both datasets exist, two related common errors appear, so-called *covariance shift* (the distribution changes between trained and testing data) and *dataset shift* (different distribution of the outputs and inputs from the test dataset regarding the distribution from the training dataset) [23]. Moreover, many ML approaches hold huge computational complexity, such as tuning learning parameters that may affect the model’s robustness.

In this research, senseFly eBee was chosen as a UAS platform to automate the mapping at high spatial resolution using an onboard narrowband Parrot Sequoia Multispectral sensor and the the senseFly’s S.O.D.A. compact digital camera. The images were separately processed through an end-to-end photogrammetric pipeline by computing the view of each image and, subsequently, the generation of a dense and scaled 3D model of the crop and orthomosaic production. Next, the plot extraction is carried out in 2D for the multispectral imagery and in 3D point clouds for the RGB data. The multi-spectral imagery (MSI) features per plot are calculated applying the ‘Triple S’ pipeline (Statistical computing of Segmented Soybean multispectral imagery) by statistically analysing the pixel values of soybean end-members by filtering the image through k-means clustering. For RGB data, algorithms were employed to analyse height variations per plot and mesh calculations were applied to quantify canopy volume using point clouds as a photogrammetric product. Features coming from both optical sensors are extracted to perform a ML model by RF (Random Forest) and XGBoost (eXtreme Gradient Boosting), training the learning process and validating it with grain yield field measurement. Therefore, the main goal is to predict the final yield based on imagery data that will allow the selection of phenotypes for practical breeding, affording constant operational improvement and proactive management with high spatial precision.

After this brief introduction, the employed materials and the proposed methodology will be described, followed by the experimental results and analysis. To finalize, the conclusions and further studies are summarized.

## Materials

### Materials

The materials used for the data acquisition are described below:

- A GNSS device from TopCon to georeference the Ground Control Points (GCPs), Hiper V receiver. The topographic surveying was done using Real-Time Kinematic (RTK).
- A general purpose GER 1500 spectroradiometer to acquire spectral measurements of the calibration targets.
- A senseFly’s S.O.D.A. Digital Camera as an RGB photogrammetric sensor, with the following technical specifications (Table 1):

**Table 1:** Technical specifications of the senseFly’s S.O.D.A. Digital Camera

Parameter	Value
Optical sensor size	116.2 mm^2^
Image size	5742*3648 pixels
Focal length	10.6 mm
Pixel size	3 µm
- A four narrowband passive sensor (Green, Red, Red-edge and Near infrared): Parrot Sequoia Multispectral sensor. The camera specifications are detailed in Table 2. It has a global shutter to avoid problems in data processing [24] and it is self-calibrating, using the incorporated Sunshine sensor.

**Table 2:** Technical specifications of the Parrot Sequoia Multispectral sensor

Parameter	Value
Spectral range	350–2500 nm
Shooting time	0.1 s
Spectral resolution	1 nm
Field of view	25º
Pixel size	3.75 µm
Focal length	3.98 mm
Image size	1280*960 pixels
- The senseFly eBee, designed as a fixed wing UAS for application in precision agriculture with incorporated GPS, IMU and magnetometer. It has a weight of 700 g and a payload of 150 g. The digital camera on-board is controlled by the senseFly eBee autopilot during the flight.

### Experimental setup

The soybean yield trial was performed at the Agronomy Center for Research and Education (ACRE) in 2018 in West Lafayette (Indiana, USA). An alpha lattice incomplete block design with 382 recombinant inbred lines, two complete replications and 32 incomplete blocks per replication was planted [25]. Concretely, the panel includes lines from three classes of families: 16 from elite parents, 12 with diverse pedigrees, and four that are high-yielding under drought conditions. The soybean field was on a silt loam soil with a pH of 6.5. The planting was performed at 2.5 cm depth in rows 0.76 m apart to a density of 40 seed/m^2^ on May 22nd, 2018. No fertilizers or herbicides for weed control were applied. Temperatures as measured by the on-farm weather station during the growing season averaged 20.56 °C in May, 22.68 °C in June, 22.78 °C in July, 22.57 °C in August, 20.98 °C in September and 11.75 °C in October. Monthly humidity, documented by the same weather station, was 72% in May, 83% in June, 82% in July, 84% in August, 81% in September and 81% in October. The study area was 282.4*109.5 m^2^, consisting of 20 rainfed plots in vertical and 45 plots in horizontal, with different widths (6 and 8 rows). The photogrammetric flight configuration was with along-and across-track overlap of ca. 75%, adequate to Pix4D software processing. A flight altitude over the ground of 60 m for MSI (MultiSpectral Imagery) and 95 m for RGB was obtained by Sensefly software, given the camera focal and the required GSD (2 inches for MSI and 1 inch for RGB). A total of 114 MSI and 63 RGB images were used for the photogrammetric processing. For the RGB flight, the exposure time was fixed to 1/2000s and the ISO was 125. 6 GCPs were placed on the ground for scaling and georeferencing purposes, identified by hand, and measured with GNSS, using RTKNAVI software [26]. GCPs are marked as dark grey rectangles and the study area was delimitated by a black rectangle in Fig. 2.

UAS flight performed as the planning flight was designed via autonomous flying mode on June 7th 2018 (Day After Planting (DAP) 15) with the G9X sensor to get the reference point cloud from the terrain and July 23rd 2018 (DAP 61) and August 1st (DAP 70) the Sequoia and G9X sensor for the study dataset, before the seed filling phenological period, from late R4 and early R5. All the experimental results obtained below were run on a 3.6-GHz desktop computer with an Intel CORE I7 CPU and 32-GB RAM.

Plant height was checked against that of 5 fixed bars randomly placed over the study area for further analysis.

Soybean harvest was conducted on October 15th, 2018 with a small-plot research combine from Almaco. Grain Yield (GY) was performed by destructively harvesting an area of 0.5 × 0.5 m in the centre of each plot. Seed samples were processed in a drying oven at 105 °C for 48 h and later weighed. For analysis, weights were extrapolated to kg/ha and converted to 13% moisture to standardize the weight between plots. From a total of 876 plots, the mean GY value per plot was 3783.409 kg/ha with a standard deviation of 769.627 kg/ha. The minimum GY was 1915.249 kg/ha and the maximum 5422.898 kg/ha; the different quartiles reach the following value of 3442.216 kg/ha (25%), 3808.639 kg/ha (50%) and 4174.101 kg/ha (75%).

## Methods

**Fig. 1:**
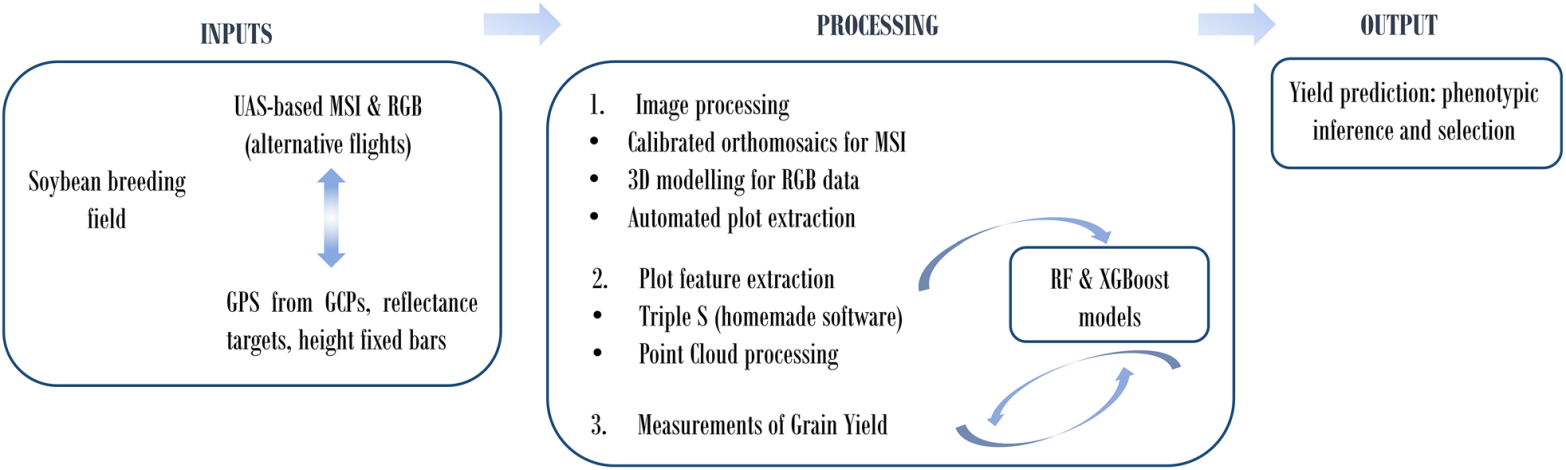
Workflow

The methodology followed is illustrated in Fig. 1. First, multispectral and RGB images are acquired by UAS over the soybean breeding field, together with measurement from height fixed bars, spectral responses from reflectance targets and GPS (Global Positioning System) data from GCPs on field. After that, a photogrammetric pipeline was carried out, obtaining orthomosaics coming from MSI and point clouds from RGB data. Features from each plot are extracted to perform a RF and XGBoost model, training the learning process and validating it with destructive grain yield measurements, with the main goal being to predict the plots grain yield based on imagery data.

### UAS imagery

Proper flight planning is crucial to guarantee the imagery acquisition reaches the theoretical parameters, produces high quality images, achieves optimization of existing resources as well as minimizes the capture time.

Once the study area is defined, Sensefly software determines the flight strips, the camera orientation and the image acquisition regarding the restricted forward and side overlap and guaranteeing the scale for the required GSD (Ground Sample Distance), 2.54 cm (1 inch) for RGB and 5.08 cm (2 inches) for MSI, based on the onboard sensor. Due to the proportion of spatial resolution of both flights, their combination in a single product is easier and there is no need for additional resampling operations. The parameters that define image capture are determined during flight execution depending on light conditions, wind and flight speed.

### Photogrammetric pipeline

Firstly, a topographic survey was performed that allows for the absolute georeferencing and scaling of the model. For this purpose, accuracy targets such as GCP were placed along the study area so as to be detectable in the acquired images. Once the aerial imagery had been captured, a standard photogrammetric pipeline was performed by image-based modelling techniques. Each dataset was handled by a framework based on camera calibration [27], image orientation and dense point cloud extraction [28]. The Pix4Dmapper software package (Pix4D SA, Lausanne, Switzerland) was employed for image processing, producing orthomosaics and 3D point clouds. In addition, the GCPs’ measurements were employed in retrieving the camera’s interior parameters and correcting for any systematic error or block deformation. At this point, it is worth mentioned that the parameter’s extraction from multispectral imagery is done through orthomosaic (i) while from RGB, geometric parameters are extracted based on 3D point clouds (ii).

i. Images gathered by the Parrot Sequoia Multispectral sensor generated datasets for each flight that included Green, Red, Red Edge and NIR information. This sensor is a radiometric self-calibrating system. It incorporates an integrated irradiance sensor (Sunshine sensor) that allows irradiance values to be synchronized with the onboard GPS, IMU and magnetometer. Moreover, the relative influence of the atmosphere is minimal because the atmospheric column spanned by the radiation is unimportant and can be neglected in the calculations [29]. To radiometrically check this calibration, at the same time to the aerial data acquisition, a radiometric campaign on field was carried out over reflectance targets. Finally, the orthomosaics for each band are accurately geo-referenced to EPSG 32616, WGS84 CRS and the bands are merged, considering the parallax, using the Geospatial Data Abstraction Library (GDAL).
ii. For the RGB data, geometric variables based on the generated point cloud, with a spatial resolution > 100 points/m^2^ and mesh calculations allows plant height estimations [4] and canopy volume, characterizing crop geometry with a high detail and accuracy (“Geometric features” section).

### Point cloud processing

Generated point clouds per each RGB flight are used to extract the soybean height and canopy volume, critical for biomass estimation [30]. In order to compute these absolute values, the reference dataset was used as explained below. These point clouds possibly enclose outliers owing to the massive and automated nature of the photogrammetric processing. To filter isolated clusters, a statistical analysis on each point’s neighbourhood is performed by assuming a Gaussian distribution of neighbors’ distances [31]. Afterward, to guarantee fully registered point clouds, the Iterative Closest Point algorithm [32] is used, getting an assumable mean error among ground points from the obtained point clouds. Afterwards, point clouds were filtered by a common bounding box, with the aim to derive physiological crop dynamics. A deviation point cloud of height variations between the reference dataset (where the plants do not emerge yet) and the studied datasets was computed. Consequently, an accurate cloud-to-cloud distance was derived, giving a local approximation model to the reference cloud by a quadric surface. These point cloud-based plant heights were calibrated by a comparison to 5 fixed bars randomly placed in the study area by measuring the height with a ruler to obtain field surveyed ground truth at the same time as the flights were performed.

The next step was the triangulation of these point clouds-based plant height. The meshing algorithm chosen was 3D Delaunay triangulation [33]. These meshes have to be refined to remove the errors generated during the automated process, through the approximation of Attene [34].

### Plot feature extraction

We extracted different features per plot grouped in radiometric (through the multispectral orthomosaic) and geometric (based on the point cloud by RGB data) parameters.

#### Radiometric features

Individual plot boundaries need to be extracted and defined separately from images with an assigned plot ID that defines their genomic type by a field-map based plot extraction. First, we created a SPH file from the field map using QGIS open source software. The script starts from the top right and builds the first polygon using the defined plot size and skips the gap between plots and generates the next one until it gets to the last plot on the bottom left. One advantage is that it can be generalized to other crop types as long as the field map is provided and the plots are planted in regular distance and have a consistent size within a trial.

Once the individual plots are extracted, the ‘Triple S’ pipeline (Statistical computing of Segmented Soybean multispectral imagery) was run. ‘Triple S’ [8] is an open source pipeline coded in Python that uses the GDAL library and Open Source Computer Vision Library [35] running over Anaconda Prompt. From each plot, it generates the following information ordered in a spreadsheet by the name of the plot file as follows: first, the image is classified in ground and soybean by *k*-means clustering [36] using the near infrared band, which provides a bigger difference in the spectral response between end-members. Once the image is filtered, the statistical parameters of the pixel-values of soybean end-member are calculated according to Gaussian and robust models. Since, the possible presence of systematisms, and/or outliers, will hinder the fulfilment of the hypothesis of a Gaussian distribution, statistics like the mean and the standard deviation will not provide a suitable analysis [37]. For this reason, the following robust estimators are adopted in the present study: the median *m*, the normalized median absolute deviation (NMAD) (Eq. 1), the square root of the biweight midvariance (BwMv) (Eq. 2), and the interpercentile ranges (IPR):

1 $$ {\text{NMAD }} = 1.4826 \cdot {\text{MAD}} $$

2 $$ {\text{BwMv}} = \frac{{n\mathop \sum \nolimits_{i = 1}^{n} a_{i} \left( {x_{i} - m} \right)^{2} \left( {1 - U_{i}^{2} } \right)^{4} }}{{\left( {\mathop \sum \nolimits_{i = 1}^{n} a_{i} \left( {1 - U_{i}^{2} } \right)\left( {1 - 5U_{i}^{2} } \right)} \right)^{2} }} $$

3 $$ a_{i} = \left\{ {\begin{array}{*{20}c} {1,if\left| {U_{i} } \right| < 1} \\ {0,if\left| {U_{i} } \right| \ge 1} \\ \end{array} } \right. $$

4 $$ U = \frac{{x_{i} - m}}{{9{\text{MAD}}}} $$

being the median absolute deviation (MAD) (Eq. 5), i.e. the median (*m*) of the absolute deviations from the data’s median (*m*_*x*_):

5 $$ {\text{MAD}} = m\left( {\left| {x_{i} - m_{x} } \right|} \right) $$

Please note that, for asymmetric distribution, will not be possible to provide a plus-minus range, therefore an absolute interpercentile range at different confidence intervals will be provided (50% also known as interquartile range, 90% and 99%), and additionally some percentile values such as 2.5%, 25%, 75% and 97.5%.

In the second step, canopy cover area (m^2^) was obtained by reading the coordinates in the metadata and relating it to the number of soybean end-member pixels. The next step consists of acquiring the number of rows through an edge map that determines if the row is completed. Canny algorithm [38] was used to obtain the edge map from the NIR band, in this case. Finally, Principal Component Analysis (PCA) [39] computes the length of each row. The row length is the number of soybean pixels along the first eigenvector of the covariance matrix [40]. Next, with median reflectance values, a bunch of VI (Vegetation Index) are calculated as Table 3 indicates:

**Table 3:** VI used as inputs from the model

VI	Equation	Proposed by
NDVI	(NIR-R)/(NIR + R)	[41]
SAVI	(1 + L)*(NIR-R)/(NIR + R+L)	[42]
MSAVI	(2*NIR + 1-((2*NIR + 1)^2^− 8*(NIR-R)*(NIR-R))^0.5^)/2	[43]
GESAVI	(NIR-a)*(R-b)/(R + z)	[44]
CIre	(NIR/RE)-1	[45]
CIg	(NIR-G)-1	[45]
VARI	(G-R)/(G + R)	[44]
RVI	(NIR/R)	[47]
DVI	(NIR-R)	[48]
RDVI	(NIR-R)/(NIR + R)^0.5^	[49]
TVI	0.5*(120*(NIR-G)-200*(R-G))	[50]

#### Geometric features

In order to extract the point cloud from each plot, the commonly used file-based solution Rapidlasso LAStools [51] was used; specifically, the tool named ‘lasclip’ using the SHP file already generated based on the field map.

Next, geometric features were extracted from the point cloud-based plant height and mesh from each plot; specifically, maximum and mean height and the standard deviation as a quantification of the height variability from the point cloud. From the mesh obtained as a triangulation of the point cloud, the canopy volume of each plot was calculated.

### ML models: RF and XGBoost

Once the plot features were extracted, the yield prediction model was performed. Specifically, machine learning algorithms develop an accurate prediction model from the training dataset. The analysis of optical sensor data often contains noise, this issue can be compensated for by adding an appropriate quantity of characteristic training data [51]. From all ML methods, assembly algorithms integrate a high number of individually weak but complementary predictors, to create a robust estimator. This amalgamation could be done as either bagging or as boosting. Furthermore, tree learning algorithms do not involve linear interactions between features (perfect for this type of data). For this study, RF as bagging and XGBoost algorithm as boosting were chosen. A brief description of these both algorithms follows.

RF is one of the most known algorithms belonging to model aggregation ideas, introduced by [52]. The basics of RF theory cover the convergence theorem and generalization error bound. More specifically, it is an ensemble machine learning method [53] based on constructing a multitude of decision trees at training time, sampled independently and with the same distribution. At each node, a given number of input variables are randomly chosen and the best split is calculated within this subset. No pruning step is performed so all the trees of the forest are maximal trees. Another advantage of RF is that it is useful not only in regression and classification problems, but also in the selection of variables. The out-of-bag (OOB) sample is the dataset not used to generate the actual tree. It is used to estimate the prediction error as well as to assess variable importance in order to perform the variable selection.

XGBoost, on the other hand, is a scalable nonlinear machine learning algorithm for tree boosting developed by [54]. This method implies a computationally effective improvement of gradient boosting decision tree implementation where a new weak learner is built to be maximally correlated with the negative gradient of the loss function related to the whole assembly for each iteration [55]. Specifically, XGBoost speeds up the boosted tree construction operating in parallel and suggests a new distributed algorithm for tree searching. The importance of each feature to the training model is considered when the boosted trees are constructed to intelligently obtain the appropriated feature scores. Another characteristic is that XGBoost additionally offer the possibility of penalizing the complexity of the trees.

To sum up, ML approaches aim to find a relationship between an input X = {x_1_, x_2_, …, x_N_} and an output Y in the training dataset and apply it to a testing dataset to assess the quality of the model. Thus, for both ML processes, *scikit*-*learn* [56] Python libraries were implemented. The study area is randomly divided into a training and a testing zone, with a range of 15% using split function imported from *sklearn.metrics* library. The random state is fixed to always obtain the same result. In the case of RF, the maximum depth of a tree was set to 5 (default 6) to decrease the complexity of the model. The number of boosted trees was set to 1000, commonly less than a thousand. For the XGBoost model, the learning rate was intentionally set to 0.06, slighter than the default value (0.3), to head up to a more precise generalization [57]. The number of boosted trees was also set to 1000 and the subsample to 0.8 to reduce the risk of over-fitting, making the training dataset more robust to the noise generating randomness. The accuracy (ACC) is calculated as follows (Eq. 6):

6 $$ {\text{ACC}} = 100 - \frac{{(100 *\mathop \sum \nolimits_{{{\text{i}} = 1}}^{{n_{test} }} \left( {\frac{{{\text{x}}_{{{\text{pred}},{\text{test}}}}^{\text{i}} - {\text{x}}_{{{\text{act}},{\text{test}}}}^{\text{i}} }}{{{\text{x}}_{{{\text{pred}},{\text{test}}}}^{\text{i}} }}} \right)^{{}} }}{{n_{test} }} $$

where $x_{pred,test}^{i}$ is the predicted GY of the *ith* plot from the testing dataset, $x_{act,test}^{i}$ is the measured GY of the *ith* plot from the testing dataset used as the actual value and n_test_ is the total number of testing samples within the study area.

## Experimental results

### MSI results by 2D image processing

**Fig. 2:**
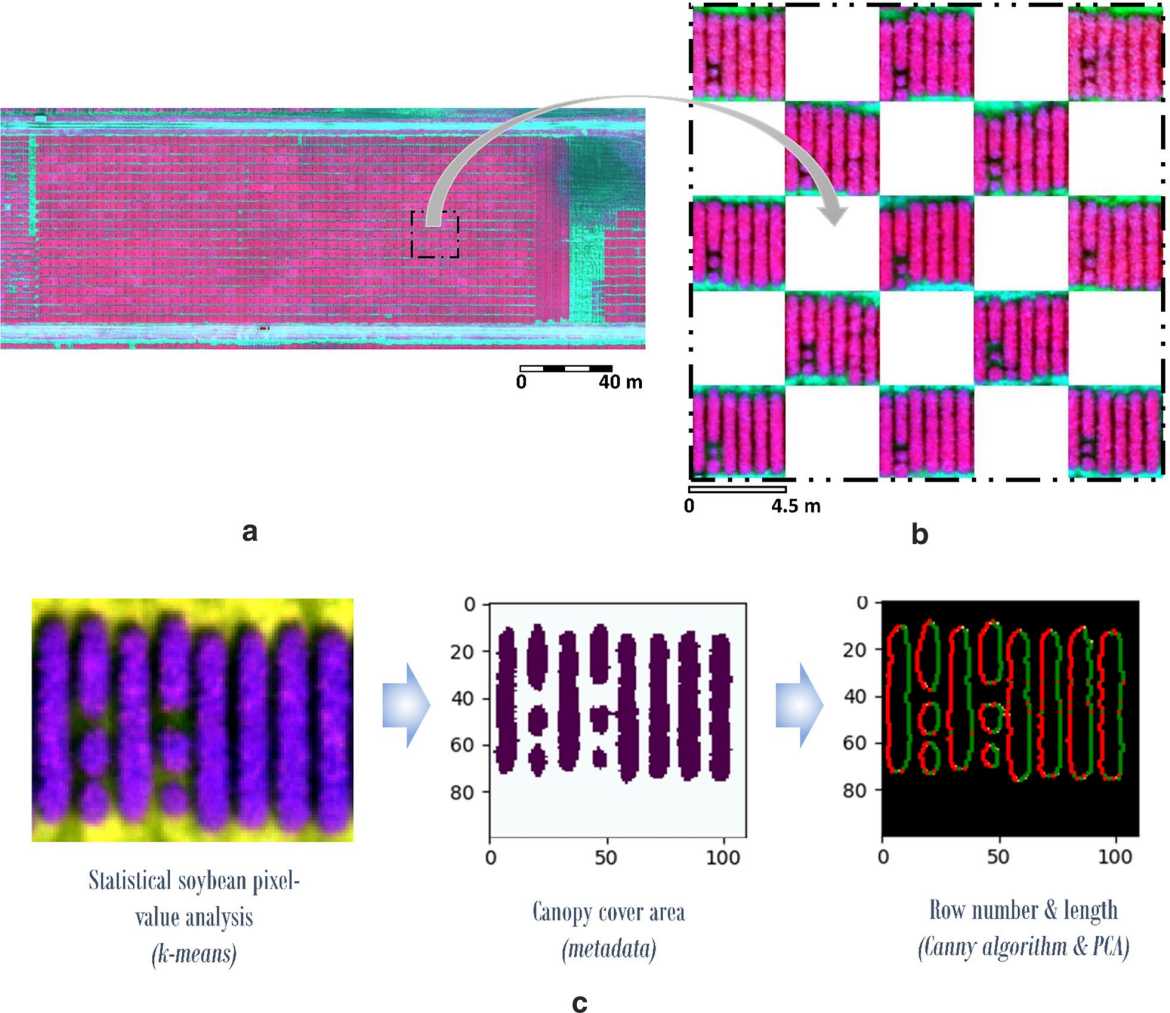
Colour composite MSI mapping over the study area on July 23rd 2018 (DAP 61) (DL (500047.3, 4480849.5); UR (500364.0, 4480968.0); EPSG 32616) **a**, a detail of field-map based plot extraction **b** and Triple S software run over a random plot for July 23rd, 2018 (DAP 61) **c**

Images gathered by the Parrot Sequoia Multispectral sensor generate datasets for each flight that included Green (G), Red (R), Red Edge (RE) and Near InfraRed (NIR) information. The weather conditions when the flights were done was clear and free of clouds (during noon time). Data was separately processed per band by a photogrammetric pipeline to obtain the orthomosaic required for GIS integration, considering the parallax. At the same time to the aerial data acquisition, a radiometric campaign on field was carried out to radiometrically check the calibration of the sensor. Thus, calibration targets were placed in the study area and measured by the spectroradiometer, obtaining a mean difference in reflectance between the measured target in field and in the orthomosaic to less than 3.02% per band. In addition, to accurately reflect the breeding field planting configuration, a script was developed to overlay defined plot sizes with known spacing and eliminate border effects by changing the plot size. This automated plot extraction allows us to analyse each plot consisting, in total, of 900 individual plots with variable size. Figure 2 illustrates the color composite of the multispectral orthomosaic (NIR + R+G) (a) and the automatic plot extraction over a randomly selected area (b). Figure 2c shows how Triple S was used for July 23rd, 2018 (DAP 61) to compute canopy cover, row number and length for one random plot. As a brief analysis, we can see how the outliers influence the values, making differences between mean and median value. The standard deviation represents the spatial variability in reflectance with no correlation found along time per band once the outliers are removed. The threshold is the value obtained using *K*-*means* (k = 2 in this case: vegetation and ground) to mask the soybean member using NIR band (band 4).

The statistics of variables from MSI analysis by plot are presented in Table 4 for the different study dates, July 23rd, 2018 and August 1st, 2018, respectively: CC (canopy cover) and soybean reflectance by band. The length of row parameter was rejected because of the lack of variation enough within the plot, also being influenced by the plot cut and the filter applied (*k*-means clustering).

**Table 4:** Statistics of canopy cover and soybean reflectance by band of soybean class per plot from MSI analysis at DAP 61 and 70: mean, standard deviation (Std), median, normalized median absolute deviation (NMAD), square root of the biweight midvariance (BwMv), percentiles at 2.5% (P2.5%), 25% (Q25%), 75% (Q75%) and 97.5% (P97.5%), interquartile range (IQR) and interpercentile range at 90% (IPR90%) and 99% (IPR99%) confidence interval

	Parameter (%)	Mean	Std	Median	NMAD	BwMv	P2.5%	Q25%	Q75%	P97.5%	IQR	IPR90%	IPR99%
7/23/2018 (DAP 61)	Canopy cover	79.54	20.29	85.45	6.66	7.60	3.37	80.09	89.47	98.32	9.37	70.54	99.85
	green	6.21	0.72	6.19	0.79	0.73	4.93	5.65	6.71	7.60	1.06	2.34	3.31
	Red	2.53	0.27	2.51	0.23	0.26	2.02	2.37	2.68	3.08	0.31	0.88	1.51
	Red edge	31.84	2.61	32.03	2.26	2.44	26.29	30.42	33.45	36.91	3.02	8.25	16.49
	Near infrared	55.15	7.02	55.42	5.80	6.83	39.45	51.70	59.48	69.23	7.78	24.46	35.83
8/01/2018 (DAP 70)	Canopy cover	86.90	5.48	87.77	3.62	3.79	69.42	85.22	90.06	94.35	4.83	19.35	31.97
	green	5.90	0.39	5.85	0.39	0.39	5.25	5.61	6.15	6.76	0.54	1.25	1.96
	Red	2.62	0.18	2.60	0.18	0.18	2.33	2.48	2.73	3.03	0.24	0.57	1.00
	Red edge	32.22	1.34	32.22	1.28	1.35	29.63	31.31	33.06	34.96	1.75	4.52	6.89
	Near infrared	55.59	2.27	55.58	2.19	2.22	50.99	54.12	57.08	60.08	2.96	7.59	13.14

It can be seen with the canopy cover parameter, the breach of the normality hypothesis causes the statistical dispersion to be overestimated, compared to robust values (NMAD, BwMv, percentile (P) and IPR).

### RGB results by 3D modelling

**Fig. 3:**
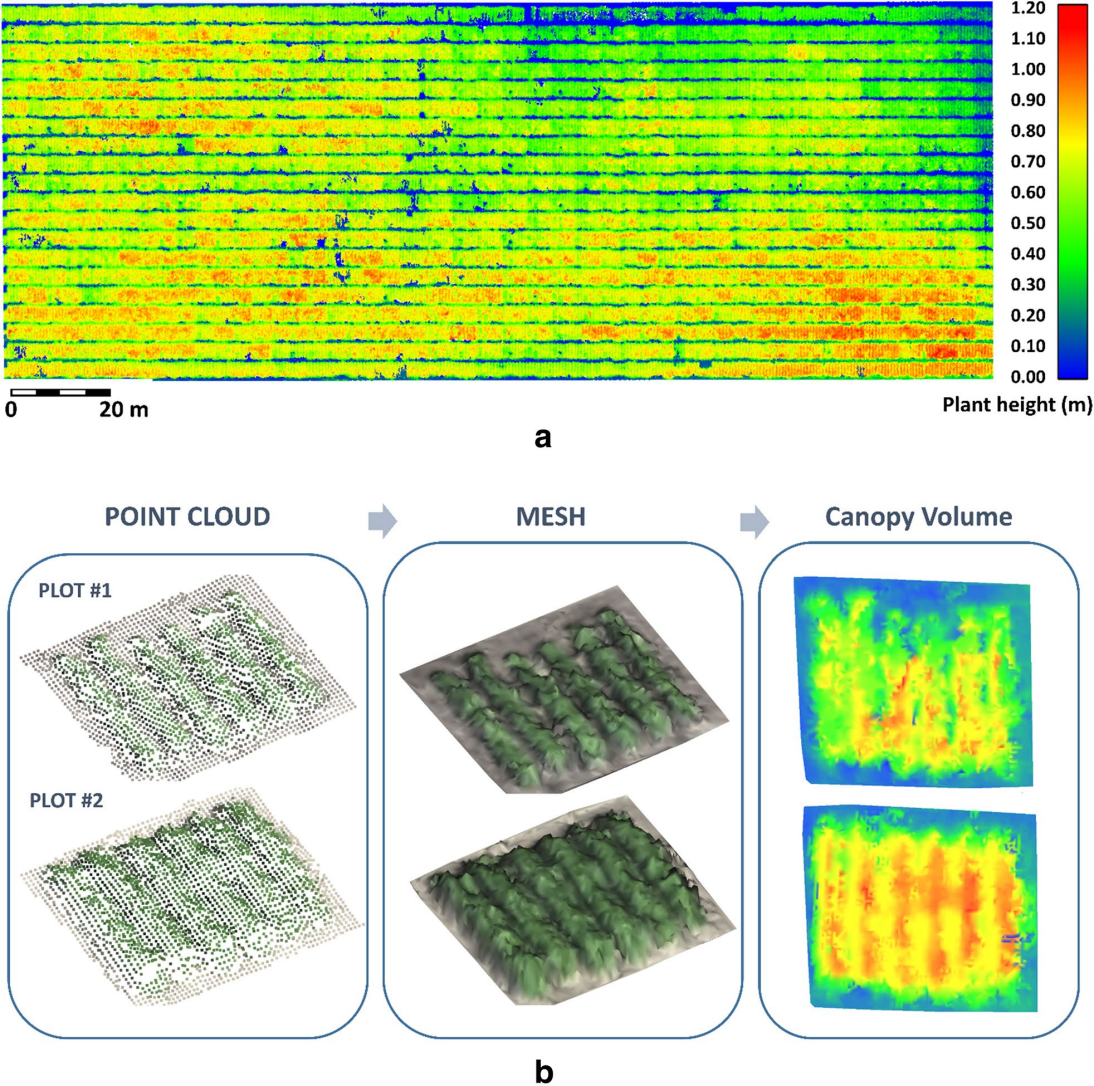
Deviation point cloud over Soybean from July 23rd (DAP 61) using June 7th (DAP 15) as reference in *meters***a** and canopy volume calculation of two random plots from the same date **b**

RGB data generates 3D point clouds. The point cloud from June 7th (DAP 15) was used as a terrain reference. It contains 1,613,588 points while the one from July 23rd (DAP 61) has 5.74% more points for the same study area, 1,711,892. The one from August 1st (DAP 70) has 1,699,878 points. Please note that the variation of the spatial resolution of the computed point clouds for DAP 61 and 70 is due to the texture changes, which affects (among other factors) the densification operation. The three flights reach the same GSD. The next step was the registration of the point cloud from DAP 61 and DAP 70 against the one from DAP 15 using the ICP algorithm [58] on terrain points. Firstly, the coarse registration was done by manually picking similar GCP. Secondly, the ICP algorithm finds that affine transformation matrix that minimizes the distances between closet points from terrain points of the two point clouds considered. Once the alignment was done, the height value was checked against 5 height fixed bars randomly placed over the study area, reaching a difference of less than 2.46 cm for the study date of July 23rd (DAP 61) and 2.21 cm for the study date of August 1st (DAP 70). On the other hand, the deviation point cloud from July 23rd (DAP 61) reaches the following statistical parameters: a minimum height of 0 m, a maximum of 1.244 m, a mean of 0.578 m and a standard deviation of 0.614 (Fig. 3a); while the one from August 1st (DAP 70) has a minimum height of 0 m, a maximum of 1.476 m, a mean of 0.798 m and a standard deviation of 0.803.

Figure 3b analyses two particular plots from July 23rd, 2018 where the visual differences in quantifying the canopy volume could be appreciated. Calibrated point clouds are converted into meshes by applying a 3D Delaunay triangulation and refined: filling of holes through algorithms of planar triangulation, repairing of meshing gaps by threshold algorithms and removal of topological and geometric noise by anti-aliased Laplacians filters. The grid was chosen as 45 cm as a trade-off between spatial resolution that affects the accuracy and computational cost. Finally, these meshes give us the value of the canopy volume per plot.

The statistics of variables from RGB analysis by plot are presented in Table 5 for the different study dates, July 23rd, 2018 and August 1st, 2018, respectively: CV (canopy volume), H max (maximum height) and variation of these parameters within the plot ( CV and  Hmax). From these results, we can affirm that these variations ( CV and  Hmax) can be assumed as equal.

**Table 5:** Statistics of CV and H max by band per plot from RGB analysis at DAP 61 and 70: mean, standard deviation (Std), median, normalized median absolute deviation (NMAD), square root of the biweight midvariance (BwMv), percentiles at 2.5% (P2.5%), 25% (Q25%), 75% (Q75%) and 97.5% (P97.5%), interquartile range (IQR) and interpercentile range at 90% (IPR90%) and 99% (IPR99%) confidence interval

	Parameter	Mean	Std.	Median	NMAD	BwMv	P 2.5%	Q 25%	Q 75%	P 97.5%	IQR	IPR 90%	IPR 99%
7/23/2018 (DAP 61)	CV (dm^3^)	1282.78	218.37	1253.85	199.04	215.52	917.37	1135.57	1413.23	1754.78	277.66	729.35	1119.80
	Hmax (cm)	92.33	16.64	87.58	10.89	12.36	73.02	81.70	97.16	139.61	15.46	56.37	84.95
	CV (% dm^3^)	19.65	4.98	19.18	4.96	4.77	11.78	15.91	22.57	31.18	6.66	16.03	27.06
	Hmax (% cm)	19.14	4.84	18.66	4.63	4.61	11.44	15.54	21.73	30.78	6.20	15.66	25.57
8/01/2018 (DAP 70)	CV (dm^3^)	1496.79	242.17	1487.83	219.58	233.32	1065.43	1327.34	1630.02	2079.86	302.69	819.91	1388.60
	Hmax (cm)	107.57	16.95	104.02	11.76	13.62	84.53	96.76	113.45	154.90	16.70	55.02	88.09
	CV (% dm^3^)	21.28	5.95	20.92	6.18	5.95	11.65	16.74	25.03	34.52	8.29	19.13	29.36
	Hmax (% cm)	20.84	5.74	20.49	5.95	5.68	11.55	16.48	24.54	33.87	8.06	18.92	30.03

In this case, the Gaussian values of the central tendency and dispersion of the parameters do not differ markedly as in the previous case (Table 6). However, the normality condition is not met in any of the previous 18 cases, with the results of the Robust Jarque–Bera test [59] for a significance level of 5%.

**Table 6:** Error metrics of both models in (kg/ha) at 95% confidence interval evaluated in training and testing dataset: MBE (Mean Bias Error), AMBE (Absolute Mean Bias Error), RMSE (Root Mean Square Error), NMAD (normalized median absolute deviation), RE (Relative Error), AE (Absolute Error) and η (the Nash and Sutcliffe index)

Dataset	Model	MBE	AMBE	RMSE	NMAD	RE	AE	η
Training	RF	13.61	140.25	181.19	167.48	1.14%	4.03%	0.80
	XGBoost	30.39	240.45	303.99	292.12	1.98%	6.87%	0.21
Testing	RF	− 4.17	325.33	410.24	384.62	1.37%	9.06%	− 2.46
	XGBoost	− 7.15	306.76	394.66	353.04	1.18%	8.55%	− 1.52

### ML model results

In this study, we developed tree learning models via RF and XGBoost for soybean yield prediction by UAS-based imagery. To sum up, we used 840 plots with a rate of 15% to check the model: 714 trained plots and 126 tested plots. The features used are 60 between both dates, 12 coming from the RGB analysis (canopy volume, maximum height and their standard deviations within each plot from DAP 61, DAP 70 and from the point cloud that represents the increment from DAP 61 to DAP 70) and 48 from the MSI coming from DAP 61 and 70, containing canopy cover value, 24 parameters from each band (mean, median, standard deviation) and 22 VI (GESAVI, NDVI, SAVI, MSAVI, CIre, CIg, VARI, RVI, DVI, RDVI and TVI). As a result, we achieve an accuracy over 90.72% by RF and 91.36% by XGBoost computed as Eq. 6 indicates.

**Fig. 4:**
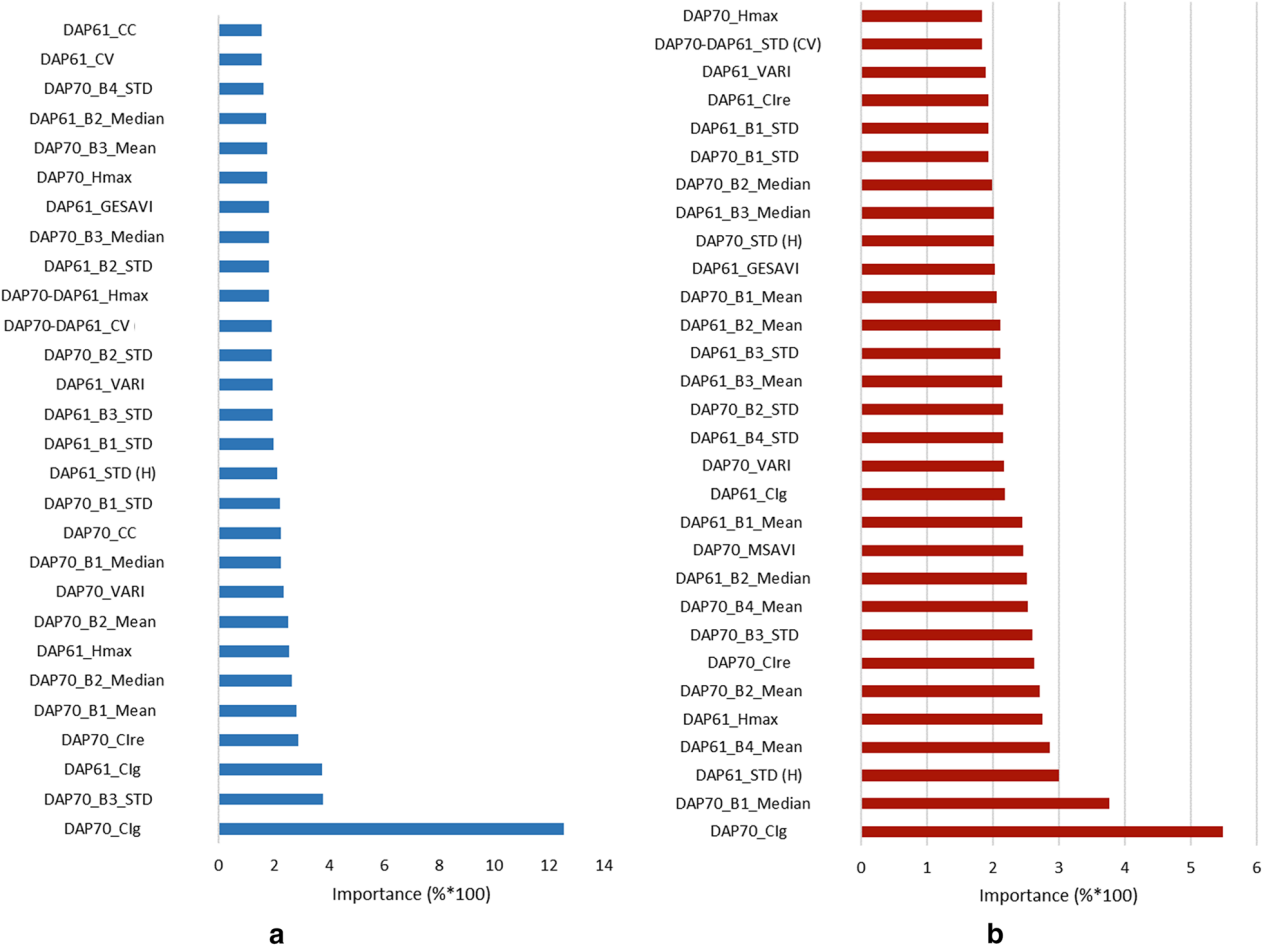
Features importance for more than 71% by RF **a** and XGBoost **b**

The features which represent more than 71% of the importance in each model are shown in the Fig. 4a by RF and Fig. 4b by XGBoost. Analysing this importance parameter, we can see that the CIg index for the DAP 70 is the most related feature while TVI and DVI are negligible regarding Grain Yield in both models. CIg represents the canopy chlorophyll content using G and NIR band.

To quantify how the sensors contribute to the accuracy of the fusion models, both models were run using only RGB features, increasing the MAE (Mean Absolute Error) in 36.99% by RF and 31.72% by XGBoost. When only MSI features are used, the MAE increases in 8.97% by RF and 14.74 by XGBoost; clearly showing how multispectral features are more related to yield than geometric measurements based on RGB data.

To analyse when the images should be captured, we run the models only with features provided by DAP 61, the MAE increases in 10.49% by RF and 12.74% by XGBoost. When the models are run with features from DAP 70, the MAE increases in 3.16% by RF and 5.95% by XGBoost. These results affirm that the images from DAP 70 better predict the yield than the images captured on DAP 61.

## Validation results and discussion

**Fig. 5:**
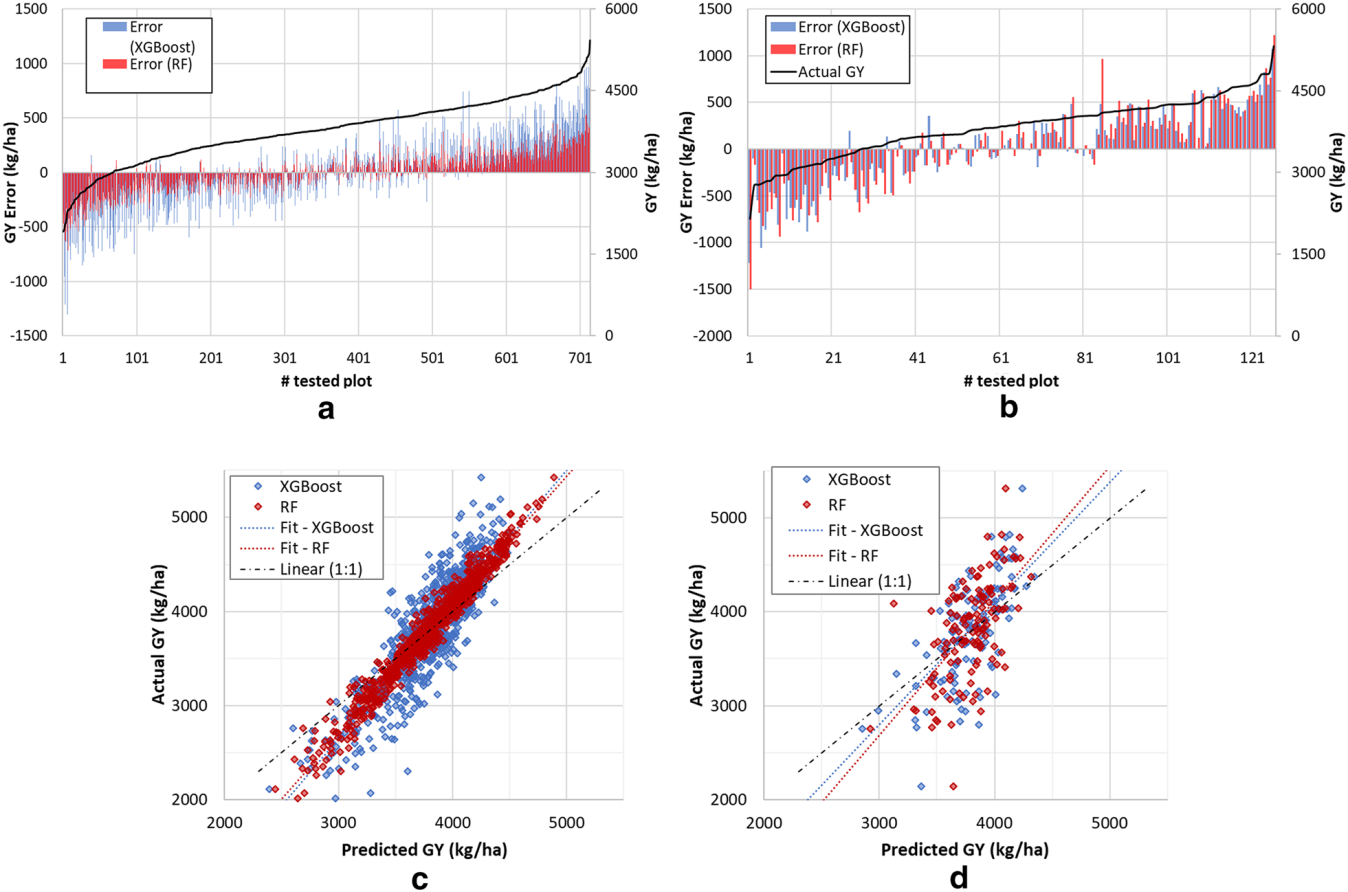
Prediction errors and actual Grain Yield (GY) sorted smallest to largest per plot along the training dataset **a** and testing dataset **b** (please note that the errors and GY are ploted in the primary and secondary axis, respectively); scatter plots of the measured against the predicted grain yield (kg*ha − 1) by RF and XGBoost from training **c** and testing dataset **d**. In both cases is drawn the line corresponding to the robust linear fit at 95% of confidence

In this section, an accurate analysis of the predicted values from the ML models is carried out. Figure 5 show the absolute errors for the actual GY sorted from smallest to largest per plot along the training dataset (Fig. 5a) and testing dataset (Fig. 5b). In both process, XGBoost and RF, the error is larger when the actual GY values are more extreme are. As expected, RF works better in fixing the training dataset than the testing, compared with XGBoost. However, we can assume that both ML approaches achive the same total accuracy generating the regression model.

Machine learning models are able to accurately fit the training data. As a disadvantage, they are susceptible to overfitting when small or large datasets with an insufficient level of variation [60]. For this reason, the validation errors along time were compared against the trained errors verifying that the validation errors do not increment while the trained errors decrease.

To quantitatively assess the models’ performance, different errors were computed. Table 6 shows the values of error metrics from both models in (kg/ha) evaluated for the training and the testing dataset. A 95% confidence level was applied to these estimated errors. As a reference value, the mean GY measured per plot is 3783.409 kg/ha for all the dataset; 3777.45 kg/ha for the training dataset and 3817.16 kg/ha for the testing dataset. The Mean Bias Error (MBE), the Absolute Mean Bias Error (AMBE), the Root Mean Square Error (RMSE), the Relative Error (RE) and the Absolute Error (AE) were computed as follows (Eqs. **7**–**11**):

7 $$ MBE = \frac{{\mathop \sum \nolimits_{i = 1}^{n} \left( {x_{pred}^{i} - x_{act}^{i} } \right)}}{n} $$

8 $$ AMBE = \frac{{\mathop \sum \nolimits_{i = 1}^{n} |\left( {x_{pred}^{i} - x_{act}^{i} } \right)|}}{n} $$

9 $$ RMSE = \sqrt {\frac{{\mathop \sum \nolimits_{i = 1}^{n} \left( {x_{pred}^{i} - x_{act}^{i} } \right)^{2} }}{n}} $$

10 $$ RE = 100*\frac{{\mathop \sum \nolimits_{i = 1}^{n} \;\left( {x_{pred}^{i} - x_{act}^{i} } \right)/x_{act}^{i} }}{n} $$

11 $$ AE = 100*\frac{{\mathop \sum \nolimits_{i = 1}^{n} |\left( {x_{pred}^{i} - x_{act}^{i} } \right)/x_{act}^{i} |}}{n} $$

where $x_{pred}^{i}$ is the predicted GY of the *ith* plot, $x_{act}^{i}$ is the measured GY of the *ith* plot used as the actual value and n is the total number of samples within the study area. The NMAD was defined in  “Radiometric features” section (see Eq. *1*).

In addition, the Nash and Sutcliffe index, η is also computed (Eq. 12); used in modelling to characterize the error related to the spatial heterogeneity:

12 $$ \eta \; = \;1\; - \;\frac{{\mathop \sum \nolimits_{i = 1}^{n} \left( {x_{pred}^{i} \; - \;x_{act}^{i} } \right)^{2} }}{{\mathop \sum \nolimits_{i = 1}^{n} \left( {x_{pred}^{i} \; - \overline{{\;x_{act}^{i} }} } \right)^{2} }} $$

where $\overline{{x_{act} }}$ is the actual average GY.

Some of these evaluation metrics have been extensively used to analysis the power of regression models [61].

Smaller values of MBE, AMBE, RMSE, NMAD, RE and AE and larger values of η (∞ < η ≤ 1) indicate better precision and accuracy of the prediction model. With these results, we can affirm that XGBoost performs better than RF for this type of data, probably dealing better with overfitting.

Figure 5 shows the scatter plots of the measured vs. predicted GY values from the training (Fig. 5c) and testing dataset (Fig. 5d) in both models, RF and XGBoost. In both cases is fit a linear function according to a bisquare weighting. For the computation the outliers are discarded according to the studentized residuals at for a significance level of 0.05 for a two tails distribution. The coefficients, the regression values (R^2^) and the highest studentized residual are shown in Table 7. The *i*-th studentized residual (*sr*_*i*_) is computed as the division of the residual (r_*i*_) of the *i*-th observation by the exact residual standard deviation [62] (Eq. 13):

**Table 7:** Robust linear fit coefficient, R^2^ value, highest studentized residuals mad RMSE & NMAD values of the fitting

Dataset	Model	a	b	R^2^	Max studentized residual	RMSE	NMAD
Training	RF	1.372	− 1420.5	0.9728	1.95	94.06	102.40
	XGBoost	1.429	− 1638.8	0.7787	1.95	262.03	263.05
Testing	RF	1.433	− 1614.7	0.3828	1.88	399.87	360.08
	XGBoost	1.290	− 1069.1	0.4183	1.89	387.11	370.03

13 $$ sr_{i} = \frac{{r_{i} }}{{\sqrt {MS_{Res} \left( {1 - h_{ii} } \right)} }} $$

being *MS*_*Res*_ the mean squared error of the regression fit calculated by removing the *i*-th observation, and *h*_*ii*_ is leverage value for the *i*-th observation (*i*-th element of the diagonal of the hat matrix).

As shown by [61], studentized residual is generally recommended instead normalized residual for least squares fit, since any point with a large residual and a large *h*_*ii*_ is potentially highly influential. If the absolute value of a studentized residuals is greater than a critical threshold, then the observation is marked as outlier. The critical threshold is defined from a *t*-distribution with *n*-*p*-1 degrees of freedom; being *n* de number of observations and *p* the number of fit coefficients. A total of 47 and 32 outliers were detected for the training RF and XGBoost models respectively; and 2 and 1 for the testing RF and XGBoost models respectively.

As we expected, both training and testing correlation (Fig. 5c, d.) shows the same tendency. The trained model under-estimates yield at high values of actual yield and over-estimates at low values of actual yield in both models. As a reason of this behaviour, we could argue that it is directly related to the distribution of the GY data regarding extreme values.

One consideration is that regarding machine learning models, the correlation coefficient R2 does not show the influence of the distribution of the training data. More values within the same range of training data will have a better prediction. R2 coefficient average all these discrepancies.

**Fig. 6:**
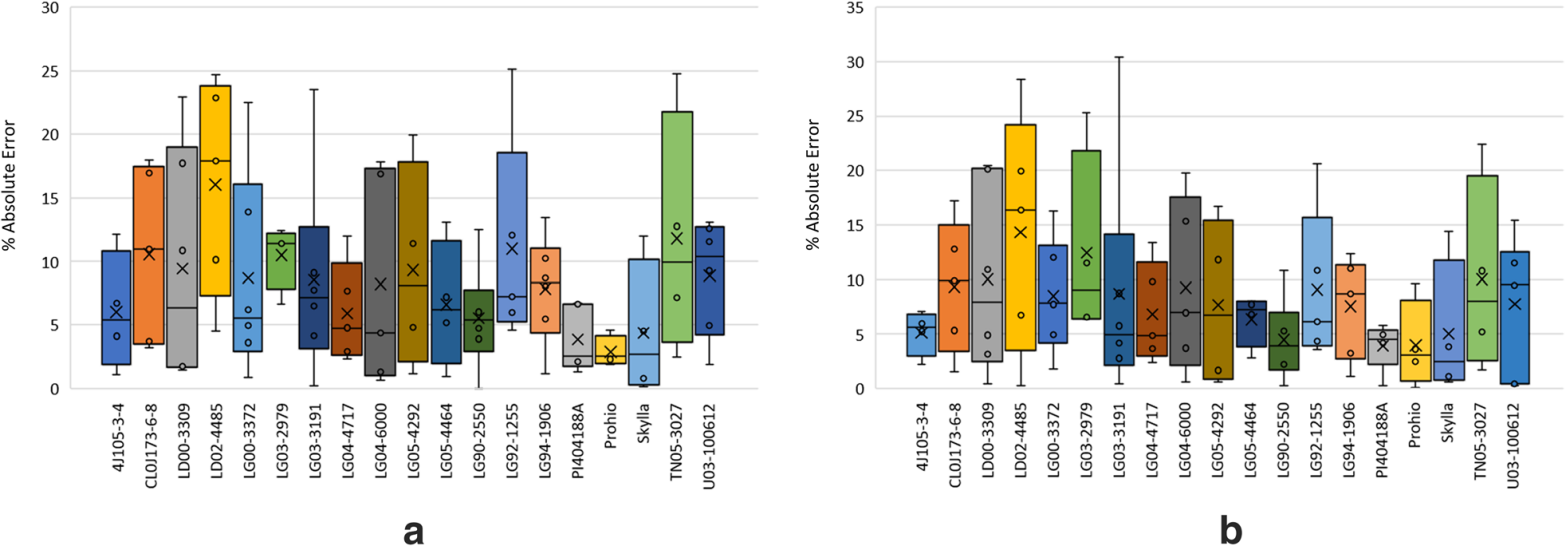
Errors from the testing dataset grouped by family: RF **a** and XGBoost **b**

A brief checkup about how different genotypes affect our GY prediction is introduced in Fig. 6, where the AE (Absolute Error) from the testing dataset is grouped by families (families within 4–6 predicted values in the testing dataset), potentially being PI404188A and Prohio the best family predicted for both models but being LG90-2550 the most consisted in both models.

## Conclusions

This paper demonstrates the great potential of UAS to predict soybean yield from multi-sensor data fusion as a rapid, accurate and cost-effective tool for automated high throughput phenotyping. Specifically, this study evaluates the power of high spatial resolution optical data, combined with regression models based on machine learning approaches (RF and XBOOST) to effectively obtain high correlations with yield in breeding trials. As a potential limitation, we found that the model has to be trained when applied due to different field conditions and soybean genotypes.

Although data fusion is able to increase the accuracy in phenotype prediction, future researches should address the efficiency of different sensor combinations. The sensor cost and the accuracy improvement should be assessed for each study. Additionally, this workflow can be successfully used for other HTPPs (High Throughput Phenotyping Platforms) and other crops planted in breeding nurseries. Even so, more comprehensive studies are necessary, including studies on different crop species at different phenotypic stages. Furthermore, UAS approaches for precision farming are in constant evolution and represents an extremely dynamic sector. In this context, this research is our contribution as a methodology for yield prediction in soybean from UAS-based multi-sensor data fusion by machine learning approaches.

## Data Availability

The datasets used and analysed during the current study are available from the corresponding author on reasonable request.
